# Recent Advances in Managing Acute Pancreatitis

**DOI:** 10.12688/f1000research.7172.1

**Published:** 2015-12-18

**Authors:** Nigeen Janisch, Timothy Gardner

**Affiliations:** 1 Department of Gastroenterology and Hepatology, Dartmouth-Hitchcock Medical Center, Lebanon, NH, USA

**Keywords:** Acute Pancreatitis, pancreatitis, Enteral Feeding, Fluid Resuscitation

## Abstract

This article will review the recent advances in managing acute pancreatitis. Supportive care has long been the standard of treatment for this disease despite extensive, but ultimately unsuccessful, efforts to develop disease-specific pharmacologic therapies. The primary interventions center on aggressive fluid resuscitation, initiation of early enteral nutrition, targeted antibiotic therapy, and the management of complications. In this article, we will detail treatment of acute pancreatitis with a focus on intravenous fluid resuscitation, enteral feeding, and the current evidence behind the use of antibiotics and other pharmacologic therapies.

## Introduction

Acute pancreatitis can be severe with extensive morbidity, mortality, and hospitalization costs ^1^. As the most common inpatient gastrointestinal diagnosis in the United States (over 270,000 inpatient stays in 2009), acute pancreatitis was responsible for USD $2.6 billion in health-care costs in 2009 ^2^. With an increasing incidence in the last decade and an overall mortality ranging from 5% to 20% depending on severity, extensive efforts have been under way to improve important clinical outcomes in the disease ^3– 5^. However, despite these efforts, no targeted pharmacologic therapy specific to acute pancreatitis has been found ^6, 7^. In this article, we will discuss advances in supportive care that have contributed to improved outcomes in this disease. In addition, we will highlight the failures of previous studies of targeted pharmacologic therapy. Finally, we will outline opportunities for future research that we feel show promise in the management of acute pancreatitis.

## Fluid resuscitation

The most effective intervention for acute pancreatitis to date is early aggressive fluid resuscitation. By providing adequate perfusion of the pancreatic microcirculation, fluid resuscitation maintains intravascular volume in the setting of the massive capillary leak associated with the inflammatory response of acute pancreatitis. In turn, preventing ischemia of the microcirculation inhibits the development of local and systemic complications such as pancreatic necrosis, systemic inflammatory response syndrome (SIRS), and multi-system organ failure ^8, 9^.

The pancreatic microcirculation can be defined as the area of vasculature, including the celiac and superior mesenteric arteries, which supplies oxygen-rich blood to the pancreatic acinar cells. Inflammatory mediators released in acute pancreatitis are thought to exert a microangiopathic effect leading to hypercoagulability with microthrombi, subsequent endothelial damage from free radical release, and finally increased capillary permeability promoting hypovolemia ^10, 11^. Disruption of the microcirculation therefore is theorized as an important factor responsible for the transition from mild, interstitial edema to severe, necrotizing pancreatitis.

The most important area of research in terms of developing targeted interventions for acute pancreatitis, in our opinion, involves fully elaborating the inflammatory cascade specific to the disease. Determining the driving stimulus behind pancreatic injury and subsequent inflammatory activation will be the critical step in designing targeted therapy.

Data from both retrospective and prospective clinical trials demonstrate that early fluid resuscitation is more effective than delayed fluid resuscitation. One recent study specifically addressed this issue by defining early fluid resuscitation as receiving greater than one third of the total 72-hour fluid volume within the first 24 hours of hospitalization and late as receiving less than one third ^12^. Although the investigation yielded no information on total infused fluid volume, they concluded that patients receiving early fluid resuscitation experienced less mortality than those receiving it late. Additional studies, including a retrospective analysis of 436 patients with acute pancreatitis which found an association between early fluid resuscitation and decreased SIRS, organ failure at 72 hours, length of hospital stay, and a lower rate of intensive care unit admission, support these conclusions ^13^.

Early fluid resuscitation is agreed upon as an intervention of paramount importance; however, to date, there are no standard guidelines on optimal fluid type or volume, rate, or duration of treatment ^14^. Human studies regarding the rate of hydration consistently show decreased morbidity and mortality with aggressive hydration in the first 24 hours, although total volume of hydration at the 48-hour mark seems to have no effect on patient outcomes. The American College of Gastroenterology guidelines currently recommend 250 to 500 mL per hour of isotonic crystalloid solution in the first 12 to 24 hours, with frequent re-evaluation every 6 hours, ultimately with the therapeutic goal of decreasing the blood urea nitrogen (BUN) level ^15^. Most experts will agree with a starting infusion of 250 to 300 mL/hour or enough to produce a urine output of at least 0.5 mL/kg, in addition to the 1- to 2-L fluid bolus given in the emergency department ^16^. The goal within the first 24 hours is a total infusion of 2.5 to 4 L, with adjustments made on the basis of the patient’s age, weight, physical exam, and comorbid conditions ^17^.

Duration of aggressive resuscitation is difficult to determine and this should be individualized. It is recommended, however, to aim for a decrease in hematocrit or BUN, or both, in the first 24 hours of hospitalization. An increased risk of pancreatic necrosis has been linked with an elevated hematocrit at admission or failure to decrease after 24 hours as well as an increase in creatinine within 48 hours in independent studies ^18– 20^. With regard to BUN, a 2011 meta-analysis of 1,043 acute pancreatitis cases showed an increased risk of mortality and death with a BUN of at least 20 mg/dL (odds ratios of 4.6 and 4.3, respectively) at admission or a rise within the first 24 hours ^21^.

The type of fluid to use for resuscitation has been incompletely studied. In the only randomized study specifically evaluating different colloid resuscitation fluids, Lactated Ringer’s solution had a greater effect on decreasing SIRS and C-reactive protein levels than normal saline ^22^.

In summary, acute pancreatitis leads to alterations in the pancreatic microcirculation brought about by an intense inflammatory cascade that has yet to be completely delineated. Aggressive fluid resuscitation is used to blunt the capillary leak syndrome associated with this cascade, although the optimal rate, type, and duration of fluid resuscitation have yet to be studied. Further studies are needed to evaluate for any complications related to over-aggressive fluid resuscitation.

**Table 1.** Summary updates for the management of acute pancreatitis.

Intervention	Recommendation
Fluid resuscitation	• Early aggressive fluid resuscitation with 250 to 500 mL/hour in the first 24 hours of admission • Use of isotonic crystalloid fluids – Lactated Ringer’s
Antibiotics	• Not recommended unless there is a documented infection • No prophylaxis for necrotizing acute pancreatitis
Feeding	• Attempt enteric feeding within the first 72 hours of admission if tolerated (can be supplemented with oral low-fat diet)
Pharmacologic strategies	• No current targeted pharmacologic therapies recommended • Rectal indomethacin 100 mg for post-procedure prophylaxis in those at high risk for post-endoscopic retrograde cholangiopancreatography pancreatitis

## Antibiotics

Given the morbidity and mortality associated with infected pancreatic necrosis, it stands to reason that giving antibiotics may serve as a solution to this problem. Pancreatic necrosis complicated by translocated enteric bacteria continues to be the most common cause of mortality in patients with acute pancreatitis that survive the early phase, accounting for up to 70% of all deaths ^4, 6^. Though still a controversial topic, prophylactic antibiotic therapy is currently not recommended to prevent pancreatic necrosis associated with acute pancreatitis ^9^.

In previous years, prophylactic antibiotics were recommended and common in practice, supported by early research showing that broad-spectrum antibiotics improved outcomes and reduced mortality ^23^. However, a 2001 study evaluated three separate randomized controlled trials comparing antibiotic prophylaxis to no prophylaxis in the setting of acute necrotizing pancreatitis. The study found reductions of 21.2% in sepsis and 12.3% in mortality in patients receiving prophylactic antibiotics; however, there was no difference in the incidence of pancreatic infection ^24^.

Studies since this report have continued to show conflicting results. A 2008 meta-analysis, including the same three previously mentioned randomized controlled trials, saw no difference in the rates of pancreatic infection or mortality between the group receiving antibiotics versus placebo ^25^. A subsequent Cochrane review confirmed no difference in mortality but found a significant difference while using imipenem alone ^26^. Most recently, in 2011, an evaluation of 14 randomized controlled trials totaling 841 patients compared those receiving antibiotics versus placebo. In the categories of mortality, incidence of infected pancreatic necrosis, non-pancreatic infection, and surgical intervention, no significant differences were reported ^27^. There may even be an association with antibiotic use and an increased risk of intra-abdominal fungal infections ^28^.

Although prophylactic antibiotics are not recommended to prevent infected pancreatic necrosis, there has been some discussion of probiotic prophylaxis with a theorized benefit through selective gut decontamination. This intervention involves giving oral antibiotics to eradicate enteric Gram-negative rods and thus reduce bacterial translocation from the gastrointestinal tract into the pancreas. A 2009 meta-analysis regarding probiotic prophylaxis resulted in no reduction in the risk of pancreatic infection or associated mortality ^29^. One large study from the Dutch Acute Pancreatitis Study group even found that in patients with predicted severe acute pancreatitis, there was increased mortality from bowel ischemia in the group given probiotics ^30^. Further studies need to be performed to assess the efficacy and safety of this possible intervention.

Ultimately, prophylactic antibiotics are not recommended for use in acute pancreatitis and should not be administered in the first 24 hours of the episode unless there is clinical suspicion for concurrent infection. Patients may present initially with sepsis, SIRS, multi-organ failure, or a combination of these and thus may have clinical symptoms such as fever that may mimic infection. Treatment with antibiotics is appropriate if after evaluation of the patient via blood cultures and fine needle aspiration of pancreatic necrosis, infection is revealed. However, if there is no obvious source of infection, antibiotics should be stopped ^15^.

## Enteral feeding

The standard of care in the past has been to maintain patients on NPO ( *nil per os*, or nothing by mouth) status until pain resolution while encouraging pancreatic rest. Currently, it is widely accepted that early enteral feeding is critical to improving outcomes ^31^. Bowel rest is associated with intestinal mucosal atrophy and increased infectious complications due to bacterial translocation. To maintain gut barrier function, enteral feeding is preferred over parenteral feeding in the management of acute pancreatitis ^32, 33^.

In mild acute pancreatitis, oral intake with a low-fat soft solid diet is often tolerated within 1 week of admission and no interventions are required. However, if the patient is not eating after 1 week, enteral feeding is recommended after cessation of nausea and vomiting, no longer requiring parenteral analgesics, reduction in abdominal pain, and return of bowel sounds ^15, 34, 35^.

In severe or predicted severe acute pancreatitis, enteral feeding is recommended to start within the first 72 hours of hospitalization with oral or tube feeding. A recent study in the *New England Journal of Medicine* demonstrated that tube or oral feedings are equivalent in terms of preventing complications ^36^. Enteral feeding is thought to preserve the enteric gut barrier to prevent bacterial translocation along with avoiding the complications associated with parenteral nutrition. A 2012 meta-analysis of 381 patients with severe acute pancreatitis confirmed the benefit of enteral versus parenteral feeds. With the groups randomly assigned to receive each variation of nutrition, those with enteral feeds benefitted in mortality, infection, and organ failure and had a lower surgical rate ^37^. Nasojejunal feeding has long been preferred, although there is evidence that nasogastric feeds have a similar effect ^38^. Although evidence shows a preference toward enteral feeding, should the patient not tolerate it or not meet nutritional goals, parenteral nutrition should be started while maintaining a slow rate of enteral feeds ^15^.

## Pharmacologic therapies

Many research initiatives have aimed at finding a targeted pharmacologic therapy for acute pancreatitis. Pharmacologic agents that initially presented the most merit were pancreatic anti-secretory agents, including somatostatin, octreotide, atropine, glucagon, and cimetidine. However, experience with these agents has been universally disappointing. For example, in 1994, a randomized controlled trial of 302 patients with acute pancreatitis treated with octreotide, a longer-acting analog of somatostatin, showed no differences in mortality or complications when compared with controls ^39^. A meta-analysis of five randomized controlled trials in 2002 showed cimetidine to be no more effective than placebo in decreasing complications or pain ^40^.

Anti-proteases, owing to their inhibition of pancreatic proteases, which could stimulate pancreatic autodigestion, were also investigated. Studies on such drugs, like gabexate mesilate, nafamostat, and aprotinin, have not consistently demonstrated therapeutic benefit and are not universally employed ^41– 44^. Platelet-activating factor antagonists such as lexipafant, antioxidants, corticosteroids, nitroglycerin, anti-interleukin-10 (anti-IL-10) antibodies, and anti-tumor necrosis factor-alpha (anti-TNF-α) antibodies have been shown to be of no value in the treatment of acute pancreatitis.

Thus, despite initial promise for many agents, there unfortunately continues to be no adequate targeted pharmacologic option with any proven benefit in randomized clinical trials ^15^. The only exception has been in the treatment of post-endoscopic retrograde cholangiopancreatography (post-ERCP) pancreatitis. In a recent multi-center, double-blind, randomized placebo controlled trial of 602 patients, there was a significant reduction in post-ERCP pancreatitis when high-risk patients received rectal indomethacin ^45^. Clinical trials gleaned similar results with rectal diclofenac ^46^. Therefore, in high-risk patients only, 100 mg of rectal indomethacin is reasonable as prophylaxis ^15^.

## Conclusions

Acute pancreatitis is a devastating disease affecting millions of people worldwide. Despite improvements in supportive care, there is currently no targeted pharmacologic therapy that is used specifically to treat this disease. Medications such as anti-secretory agents and anti-proteases have been studied and failed to improve clinical outcomes. On the horizon, the key to improving outcomes in acute pancreatitis will be to develop therapies that specifically target the immune storm caused by pancreatic autodigestion. Specific immunologic therapies that target specific responses in the disease will be the key to its control.

## Abbreviations

BUN, blood urea nitrogen; ERCP, endoscopic retrograde cholangiopancreatography; NPO, *nil per os*; SIRS, systemic inflammatory response syndrome.
